# Frontiers and hotspots evolution of machine learning in gout from 2004–2023: A bibliometric analysis

**DOI:** 10.1097/MD.0000000000051366

**Published:** 2026-10-23

**Authors:** Xu-ming Zhang, An Wu, Fang Liu, Sen-guo Ji

**Affiliations:** a JiuJiang No.1 People’s Hosptial, Jiujiang, Jiangxi, China.

**Keywords:** bibliometric, gout, machine learning

## Abstract

**Objective:**

This study aimed to explore the evolution of frontiers and hotspots in machine learning for gout from 2004 to 2023.

**Methods:**

The database of publications was sourced from the Science Citation Index-Expanded within the Web of Science Core Collection. Employing CiteSpace 6.2.R5 software, a visual analysis of the core information within these publications was conducted. This retrieval spanned from 2004 to 2023 and was completed within the confines of a single day, specifically on October 20, 2023.

**Results:**

In total, 219 articles were eventually included in the analysis. Publication activity remained limited before 2018 but increased markedly thereafter; 47 articles were published in 2023 by the search date of October 20. In terms of citation impact, Breiman L holds the most significant influence. China and the United States of America, along with their affiliated academic institutions, exert substantial academic influence in this field, surpassing other countries and institutions. “Ascorbic acid” is the keyword with the longest burst duration, and “prediction” holds the highest strength. The cited journals encompass various disciplines, including internal medicine, cardiology, analytical chemistry, and endocrinology and metabolism. The prevailing developmental pattern in the field of machine learning in gout primarily revolves around interdisciplinary integration.

**Conclusion:**

Machine learning has indeed become a frontier and hotspot in aiding the prediction and diagnosis of gout over the past 5 years. The acceptance of predictive diagnostic models and the determination of the most suitable algorithmic models remain the current and future research focal points in this field.

## 1. Introduction

Gout is a common and prevalent ailment within the field of rheumatology. It is characterized by elevated serum uric acid levels, acute joint inflammation, and recurrent attacks.^[1–3]^ Global epidemiological studies have revealed that the incidence of gout is as high as 6.8%, with a higher prevalence in males, and the rates are on an upward trajectory.^[4,5]^ It is estimated that approximately 1.3 million new cases of disability due to gout occur each year.^[6]^ Owing to the often covert nature of the disease in its early stages, most patients only seek diagnosis and treatment when acute joint pain disrupts their daily lives.^[7]^ Furthermore, gout can lead to damage in the heart, cardiovascular system, and kidneys.^[8–11]^ Despite effective management of the condition through urate-lowering therapy^[12,13]^ and dietary measures,^[14,15]^ the disease continues to manifest in recurrent attacks due to issues related to patient compliance.^[16,17]^ Consequently, the development of high-tech predictive diagnostic models for monitoring gout relapse has become a prominent focus in the academic sphere in recent years.

Machine learning, by virtue of its ability to construct rapid and efficient algorithms and models, allows for the prediction and diagnosis of disease progression without the need for inherent costs, time, and risks.^[18–21]^ Hence, machine learning has emerged as one of the most prominent areas of research in the medical community today.^[22,23]^ Given the challenges of low patient compliance and high recurrence rates among gout sufferers, the utilization of machine learning to aid in the construction of diagnostic predictive models has become an urgent necessity for frontline clinical practitioners. Multiple studies have demonstrated that machine learning can assist clinicians in identifying and predicting gout-related biomarkers. For instance, Shen^[24]^ and Wang^[25]^ have utilized serum metabolomics features to detect or predict the progression of hyperuricemia at an early stage. Jiang^[26]^ has also used this method to predict the progression of coronary heart disease complications in gout patients. However, there is still a notable scarcity of bibliometric research concerning machine learning in the field of gout.

CiteSpace software (Drexel University), as a tool for visual analysis, adeptly elucidates both qualitatively and quantitatively the intricate details of publications, including the participating nations, key institutions, principal authors, and their complex collaborative networks.^[27,28]^ This bibliometric analysis presents a richer tapestry of publication information compared to traditional literature studies.^[29–31]^ Although individual studies have evaluated machine learning models for gout-related biomarkers, diagnosis, and complications, the field lacks a systematic bibliometric synthesis integrating its publication trajectory, leading contributors, collaboration structure, co-citation foundations, and evolving research themes. To address this gap, we used CiteSpace to map the knowledge structure and thematic evolution of machine learning research in gout from 2004 to 2023. This study therefore provides a field-level overview that complements, rather than duplicates, conventional reviews of individual algorithms or clinical applications. The purpose of this study was to conduct a comprehensive bibliometric analysis of the knowledge structure, research hotspots, and thematic evolution of machine learning in gout over nearly 2 decades, from 2004 to 2023.

## 2. Material and methods

### 2.1. Data sources and search strategy

In this study, all data were derived from the Science Citation Index-Expanded (SCI-E) within the Web of Science Core Collection (WoSCC). Figure 1 and Table 1 display the data retrieval strategy flowchart, primarily focusing on the 2 key themes: “gout” and “machine learning.” The search encompassed publications spanning nearly 20 years (October 21, 2004, to October 20, 2023) and was completed within a single day on October 20, 2023. Only papers published in English and categorized as articles were included, without any restrictions on the country of publication. A total of 219 articles were identified, which were exported from the online database, formatted, named “download_**.txt,” and then imported into CiteSpace 6.2.R5 for duplicate removal, ensuring no repetitions. SCI-E within WoSCC was selected because it provides standardized bibliographic and cited-reference metadata and a consistent full-record export format compatible with CiteSpace, which facilitates reproducible co-citation and network analyses. However, the use of a single database may exclude relevant publications indexed only in other sources; this limitation is addressed below.

**Table 1:** The topic search query (WoSCC < October 21, 2004 to October 20, 2023 >).

Set	Results	Search query
#1	43,053	TS = ((gout) OR (gouty attacks) OR (hyperuricemia) OR (urate) OR (uric acid))
#2	640,672	TS = ((machine learning) OR (deep learning) OR (artificial intelligence) OR (machine intelligence) OR (neural network) OR (natural language processing) OR (hybrid intelligent system) OR (CNN) OR (LSTM) OR (RNN))
#3	219	#1 AND #2 and Article (document types) and English (languages)

WoSCC = Web of Science Core Collection.

**Figure 1.**
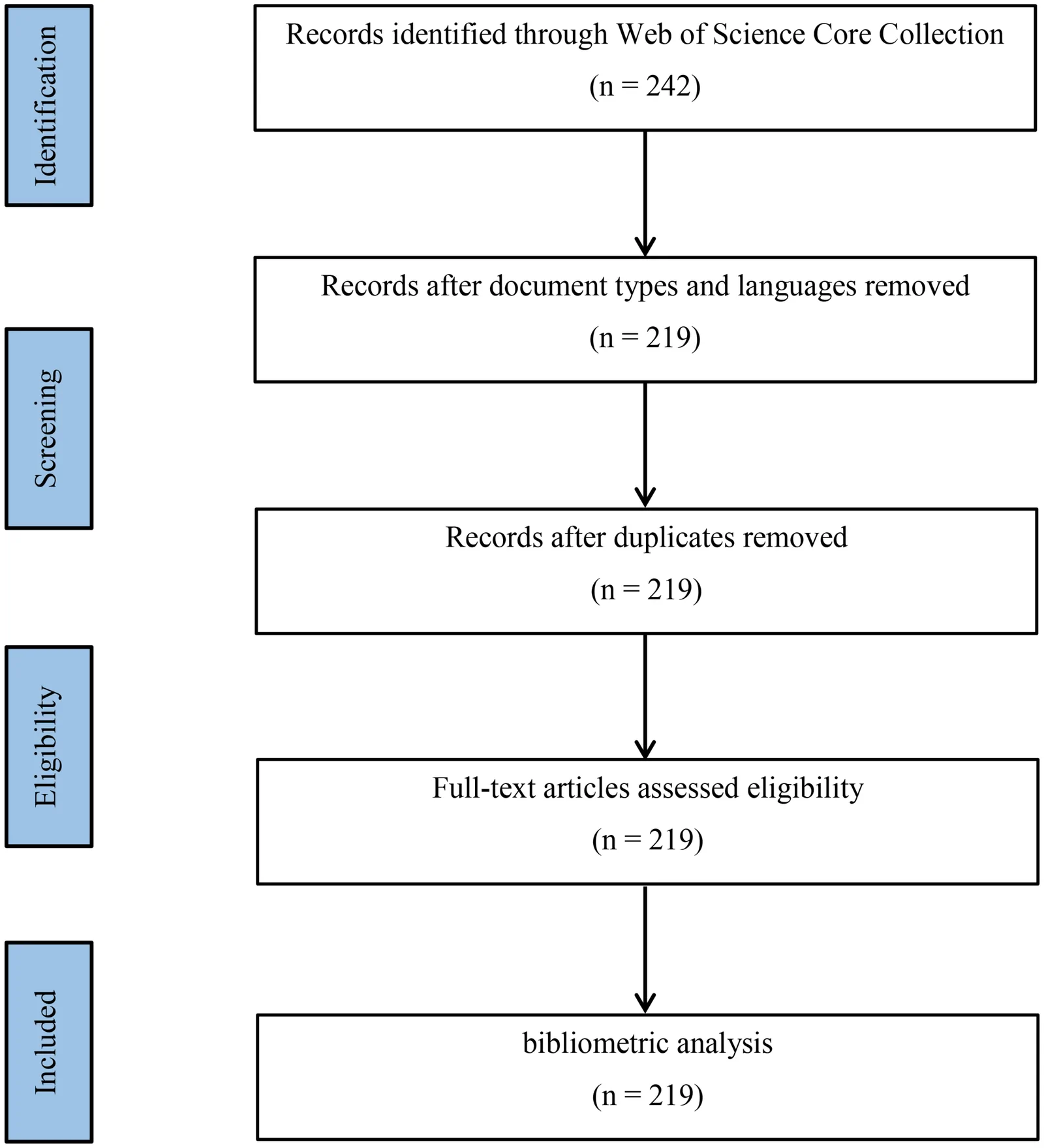
Map of literature screening process related to machine learning in gout. n = number of records.

### 2.2. Analysis tool

We utilized CiteSpace 6.2.R5 as our visualization analysis tool. Our generated charts typically provide a comprehensive visual analysis of information on countries, academic institutions, authors, journals, and keywords. Interpreting these charts aids in identifying the hot topics in machine learning applications in gout.

In the CiteSpace networks, nodes represent the analyzed entities (authors, cited authors, countries, institutions, cited journals, or keywords), and links represent collaboration, co-citation, or co-occurrence, as appropriate. Node size reflects occurrence or citation frequency. Betweenness centrality quantifies the extent to which a node lies on the shortest paths connecting other nodes; higher values indicate a stronger bridging role within the generated network. Authors and institutions were ranked primarily by publication count, cited authors and journals by co-citation frequency and centrality, and countries by publication count and centrality. Research themes were identified from high-frequency/high-centrality keywords, burst keywords, and keyword timeline clusters. These indicators describe prominence and structural position within the retrieved dataset and should not be interpreted as direct measures of research quality or clinical importance. The analysis used 1-year time slices (2004–2023), g-index k = 50, and Pathfinder pruning.

## 3. Results

### 3.1. Annual publications and trends

Figure 2 vividly displays the annual distribution of published articles. The results reveal a total of 219 articles aligning with the focus of this study. Prior to 2008, there were no articles related to machine learning in gout. From 2008 to 2017, the annual output of publications remained noticeably low. It wasn’t until 2018 that the number of publications began to show exponential growth. As of October 2023, the annual publication output has reached 47 articles, and considering the growth trajectory, the year 2023 is expected to set a new record high in publication numbers.

**Figure 2.**
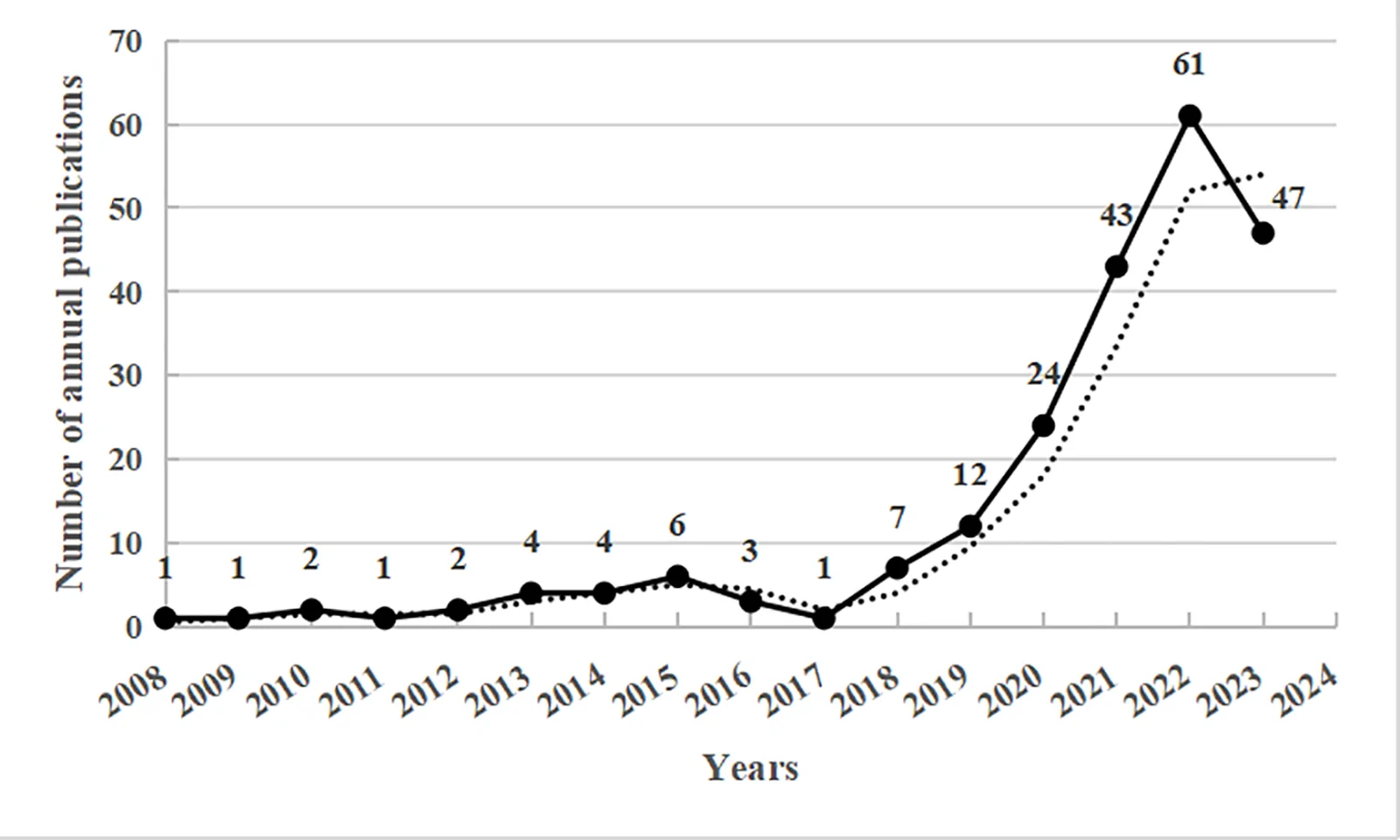
Map of annual publications related to machine learning in gout.

### 3.2. Analysis of authors and cited authors

Figure 3 illustrates a collaborative network among authors, comprising 419 nodes and 976 edges. Due to the relatively late onset of machine learning applications in clinical settings, as of October 2023, a core group of authors has yet to emerge in the field of machine learning for gout. At this juncture, Chen, Huiling, and Daudon, Michel share the top spot for the number of publications. Nevertheless, each of these authors has only contributed a total of 3 articles, which is insufficient to establish them as central figures in this field (Table 2). Furthermore, among the top 10 authors, 4 originate from China, indicating that in the coming years.

**Table 2:** Top 10 authors related to machine learning in gout.

Rank	Author	Frequency	Year	Country
1	Chen, Huiling	3	2015	Peoples R China
2	Daudon, Michel	3	2022	France
3	Jalalvand, Ali R	2	2014	Argentina
4	Gholivand, Mohammad-Bagher	2	2014	Iran
5	Surareungchai, Werasak	2	2021	Thailand
6	Capra, John A	2	2022	USA
7	Sun, Nianrong	2	2022	Peoples R China
8	Deng, Chunhui	2	2022	Peoples R China
9	Bejan, Cosmin	2	2022	USA
10	Zhu, Jie	2	2021	Peoples R China

USA = United States of America.

**Figure 3.**
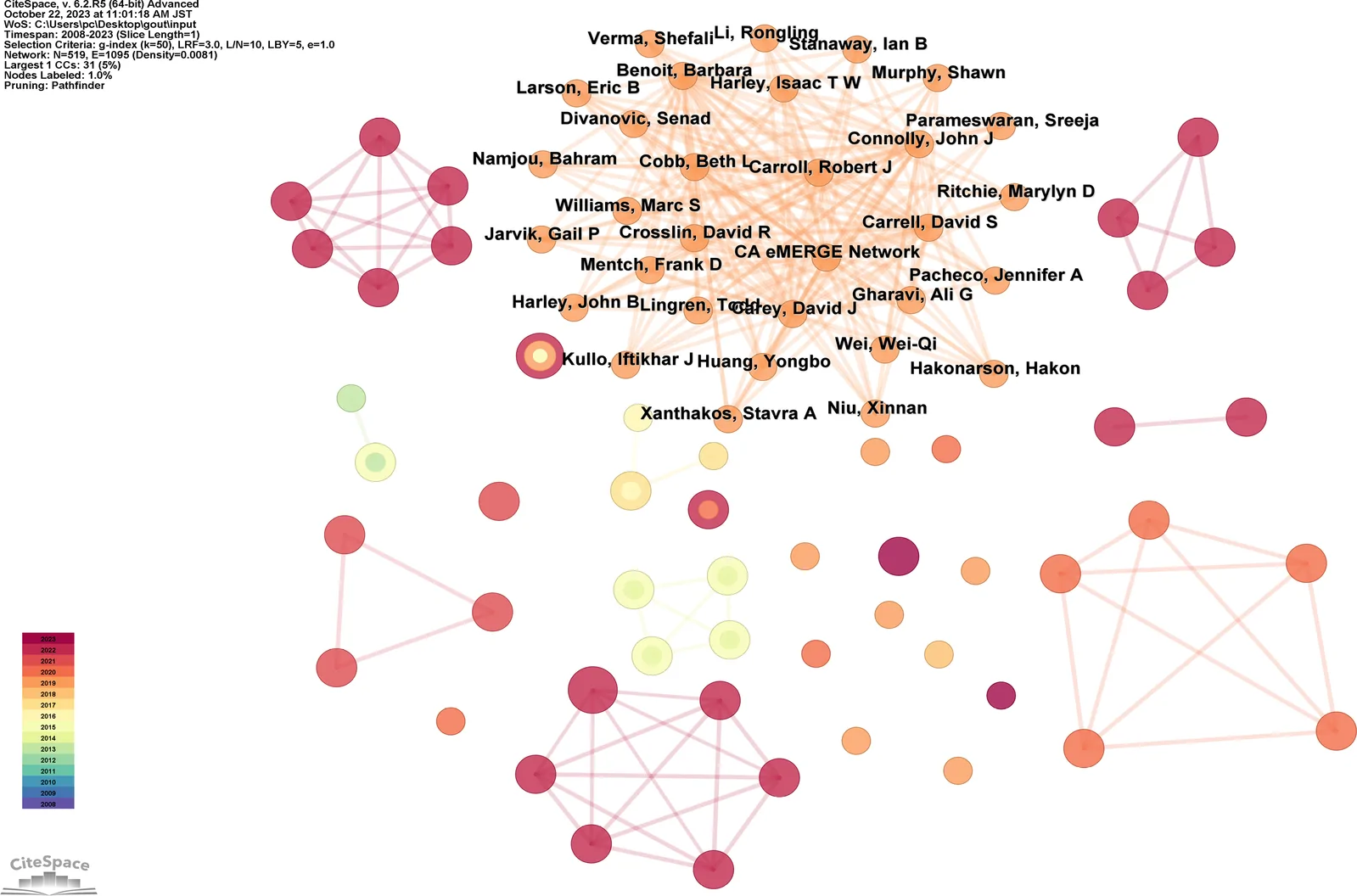
Map of authors related to machine learning in gout.

Figure 4 depicts a collaborative network among cited authors, consisting of 499 nodes and 1639 edges. Chen TQ had the highest citation count (17 citations), followed by Breiman L (16), Liu Y (12), Levey AS (11), and Lundberg SM (10) (Table 3). Among these highly cited authors, Breiman L holds the top rank in centrality, significantly surpassing other highly cited authors. Furthermore, the articles authored by Breiman L have been cited only one less time than the top-cited article. This underscores the substantial reference value of Breiman L contributions to the field of machine learning in gout.

**Table 3:** Top 10 frequency and centrality of cited authors related to machine learning in gout.

Rank	Frequency	Author	Rank	Centrality	Author
1	17	Chen TQ	1	0.37	Breiman L
2	16	Breiman L	2	0.08	Friedman JH
3	12	Liu Y	3	0.08	Chen Y
4	11	Levey AS	4	0.06	Ke GL
5	10	Lundberg SM	5	0.05	Coresh J
6	9	Pedregosa F	6	0.05	Chen X
7	8	Chawla NV	7	0.04	Black KM
8	8	Lee S	8	0.04	Li Q
9	7	Choi HK	9	0.04	Hall M
10	7	Wang Y	10	0.04	Kononenko I

**Figure 4.**
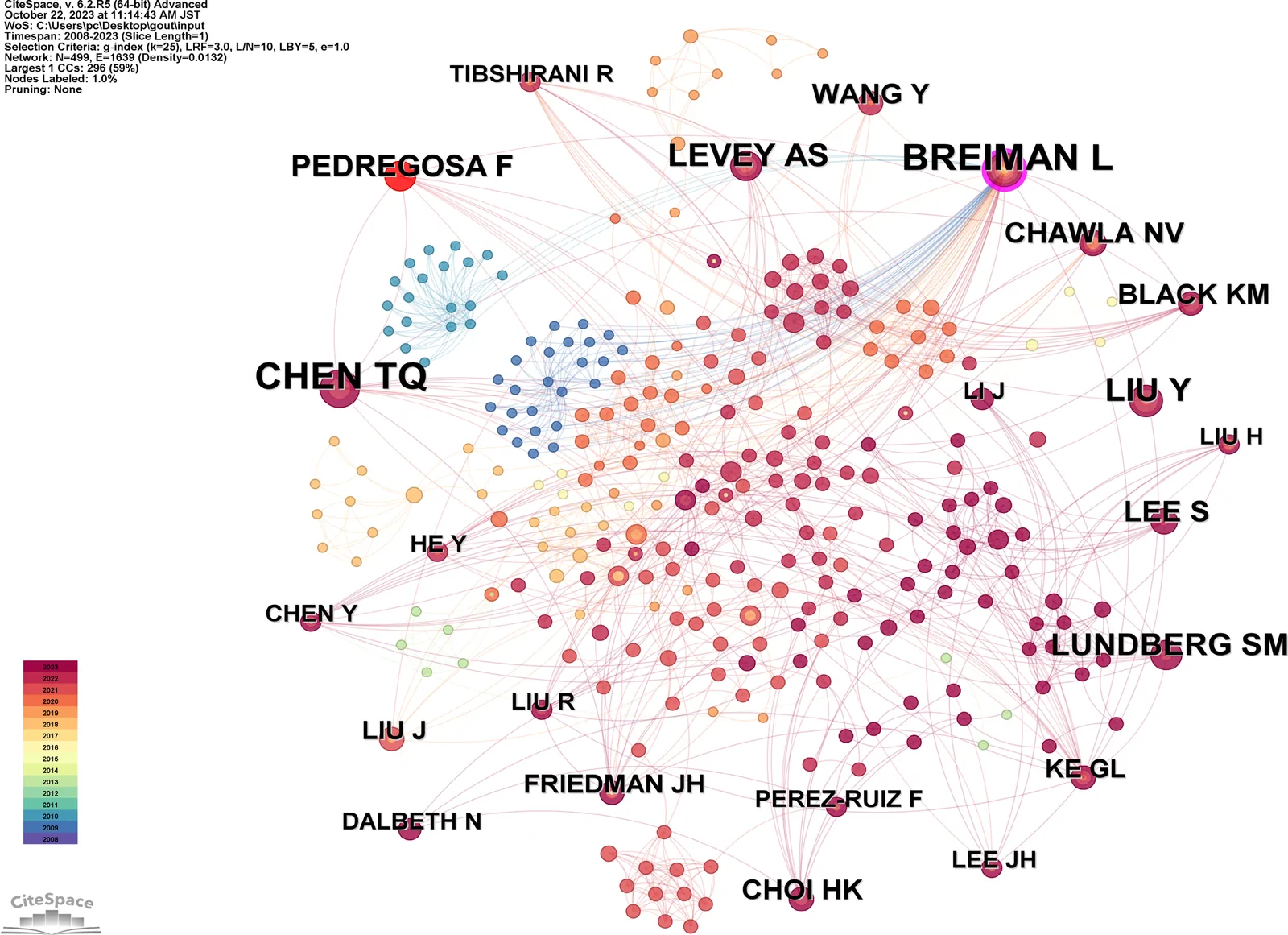
Map of cited authors related to machine learning in gout.

### 3.3. Analysis of countries and institutions

Figure 5 and Table 4 display a collaborative network among nations, formed by 52 nodes and 110 links. The results indicate that China (with 105 published articles and a centrality of 0.34) and the United States (US) (with 52 articles and a centrality of 0.84) have significantly higher academic influence in this field than other countries. However, while China’s total publication output exceeds that of the US by 101.92%, its centrality lags behind the US by 147.06%.

**Table 4:** Top 10 frequency and centrality of countries related to machine learning in gout.

Rank	Frequency	Countries	Rank	Centrality	Countries
1	105	Peoples R China	1	0.84	USA
2	52	USA	2	0.34	Peoples R China
3	10	Iran	3	0.14	Germany
4	9	India	4	0.14	Australia
5	9	Germany	5	0.11	Iran
6	8	South Korea	6	0.09	England
7	7	France	7	0.09	Taiwan
8	7	England	8	0.09	Denmark
9	7	Taiwan	9	0.07	France
10	7	Japan	10	0.07	Japan

USA = United States of America.

**Figure 5.**
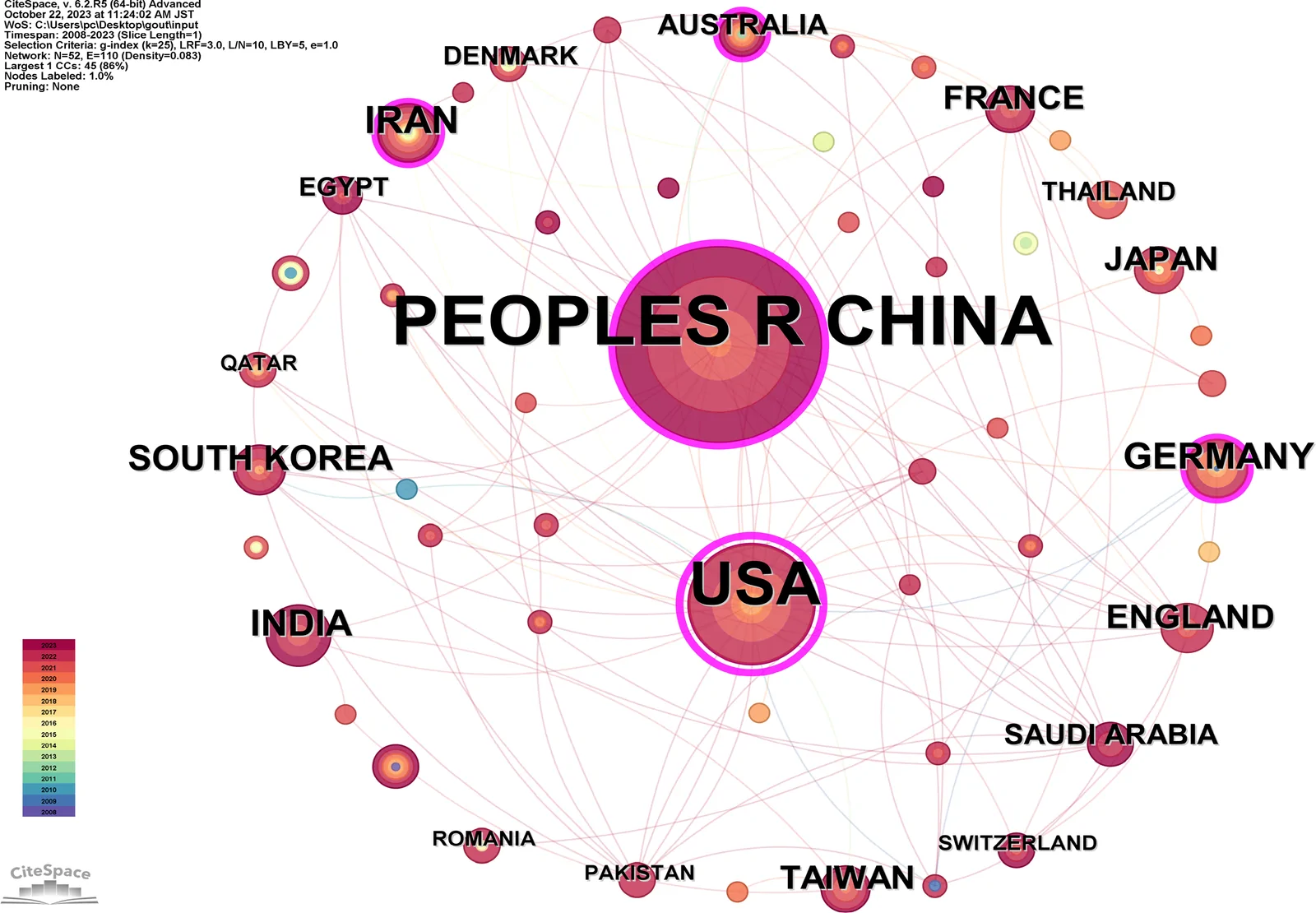
Map of countries related to machine learning in gout.

Figure 6 and Table 5 reveal a network of international collaboration comprising 217 nodes and 447 links. The top 10 institutions by publication volume are: the Chinese Academy of Sciences (9 articles), Sichuan University (7 articles), Fudan University (7 articles), Harvard University (6 articles), Peking University (6 articles), Harvard Medical School (5 articles), University of California System (5 articles), Vanderbilt University (5 articles), Sun Yat-sen University (5 articles), and Brigham and Women’s Hospital (5 articles). Notably, the top 3 positions are occupied by Chinese institutions. Moreover, the top 10 academic institutions, located in either China or the US, have broad collaboration networks with other countries, highlighting their central academic influence in the field of gout and machine learning. However, considering the global prevalence of gout, the 10 most productive institutions were located in China or the US, and the network visualization showed collaboration links involving these institutions.

**Table 5:** Top 10 publications of institutions related to machine learning in gout.

Rank	Frequency	Year	Institutions
1	9	2018	Chinese Academy of Sciences
2	7	2018	Sichuan University
3	7	2020	Fudan University
4	6	2019	Harvard University
5	6	2016	Peking University
6	5	2019	Harvard Medical School
7	5	2021	University of California System
8	5	2013	Vanderbilt University
9	5	2020	Sun Yat-Sen University
10	5	2019	Brigham & Women’s Hospital

**Figure 6.**
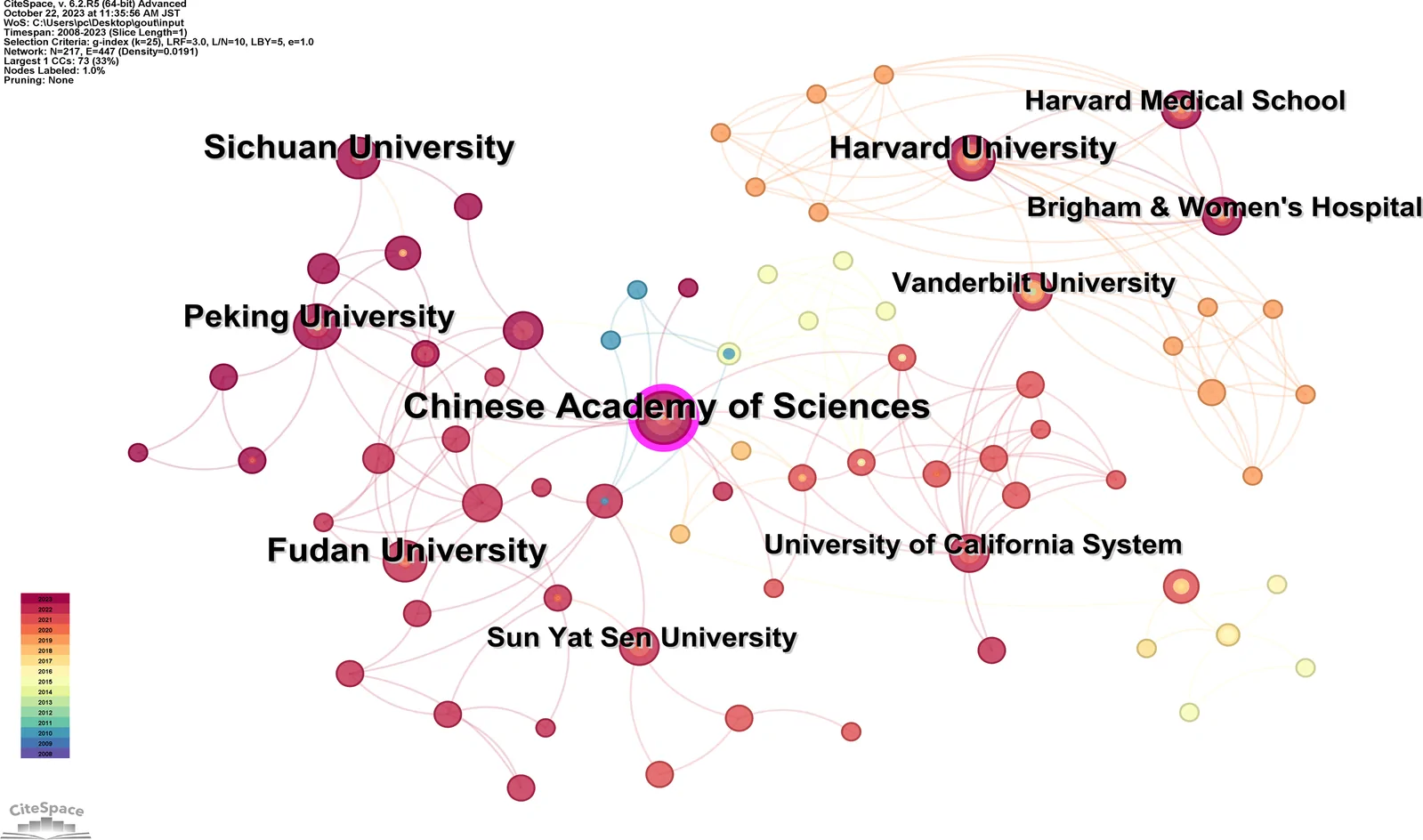
Map of institutions related to machine learning in gout.

### 3.4. Analysis of cited journals

Figure 7 and Table 6 provide the cited journals in the field of machine learning in gout from 2004 to 2023. The top 10 cited journals are as follows: PLOS ONE (81), LANCET (58), SCI REP-UK (53), NEW ENGL J MED (51), CIRCULATION (45), JAMA-J AM MED ASSOC (45), ANN INTERN MED (33), ANAL CHEM (33), DIABETES CARE (28), and KIDNEY INT (28). These journals encompass a broad spectrum of disciplines, including internal medicine, cardiology, analytical chemistry, and endocrinology and metabolism.

**Table 6:** Top 10 frequency and centrality of cited journals related to machine learning in gout.

Rank	Frequency	Cited Journals	Rank	Centrality	Cited Journals
1	81	PLOS ONE	1	0.53	ANAL CHEM
2	58	LANCET	2	0.19	AM J KIDNEY DIS
3	53	SCI REP-UK	3	0.16	ANAL CHIM ACTA
4	51	NEW ENGL J MED	4	0.13	BIOSENS BIOELECTRON
5	45	CIRCULATION	5	0.11	ACTA PAEDIATR
6	45	JAMA-J AM MED ASSOC	6	0.10	DIABETES CARE
7	33	ANN INTERN MED	7	0.09	BMC BIOINFORMATICS
8	33	ANAL CHEM	8	0.09	CLIN CHIM ACTA
9	28	DIABETES CARE	9	0.08	JAMA-J AM MED ASSOC
10	28	KIDNEY INT	10	0.07	PLOS ONE

**Figure 7.**
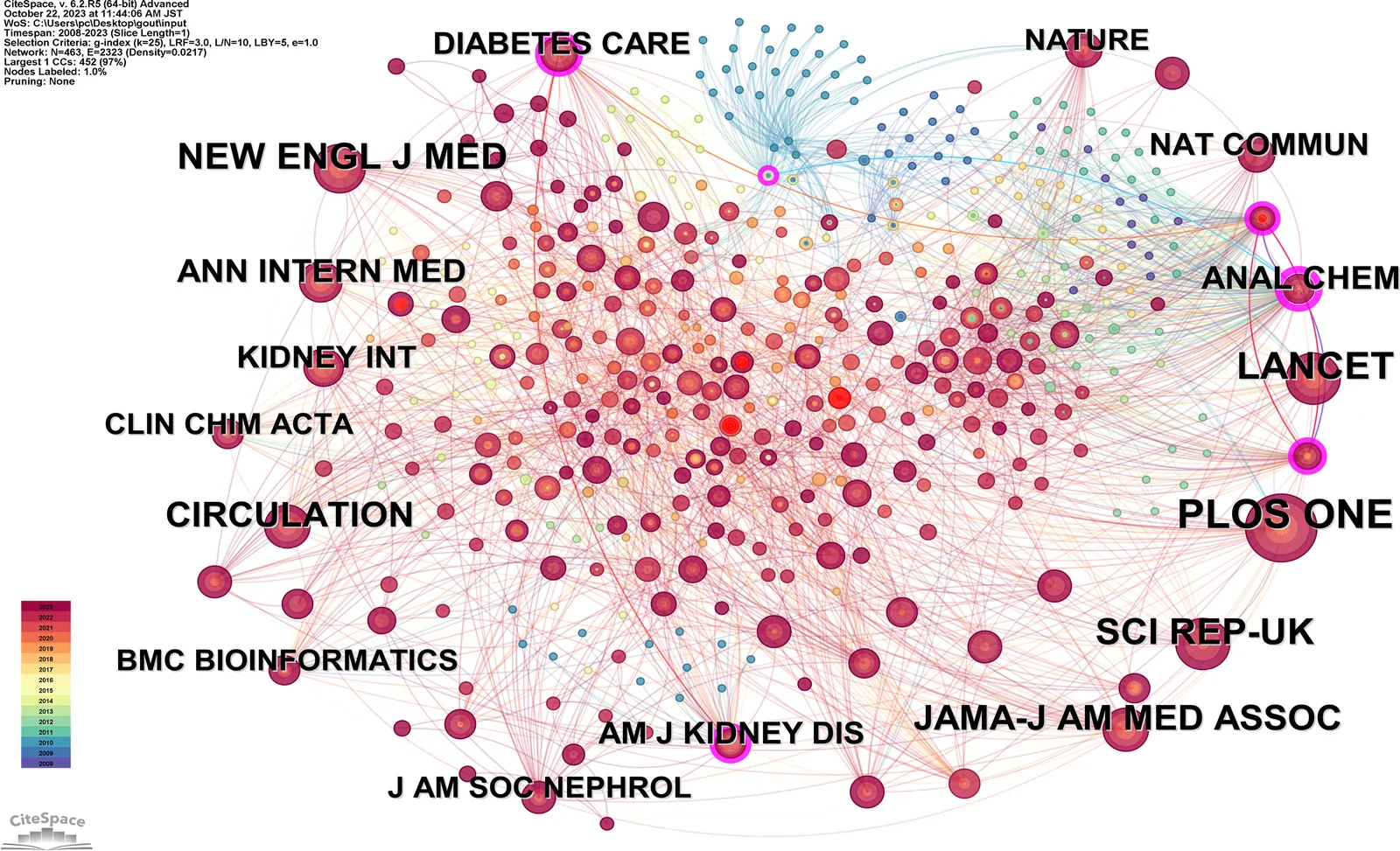
Map of cited journals related to machine learning in gout.

### 3.5. Analysis of dual-map overlay of the references

Figure 8 presents a bibliographic map illustrating the disciplinary development relationships within the field of machine learning in gout. On the left are the citing journals, representing the origins of the discipline’s development, and on the right are the reference journals, symbolizing the current state of disciplinary progress. A clear observation from the examination of the dual-map overlay is that the green and orange main threads are formed by the convergence of 2 distinct fields. Consequently, it can be inferred that the development pattern in the field of gout and machine learning is centered around interdisciplinary integration.

**Figure 8.**
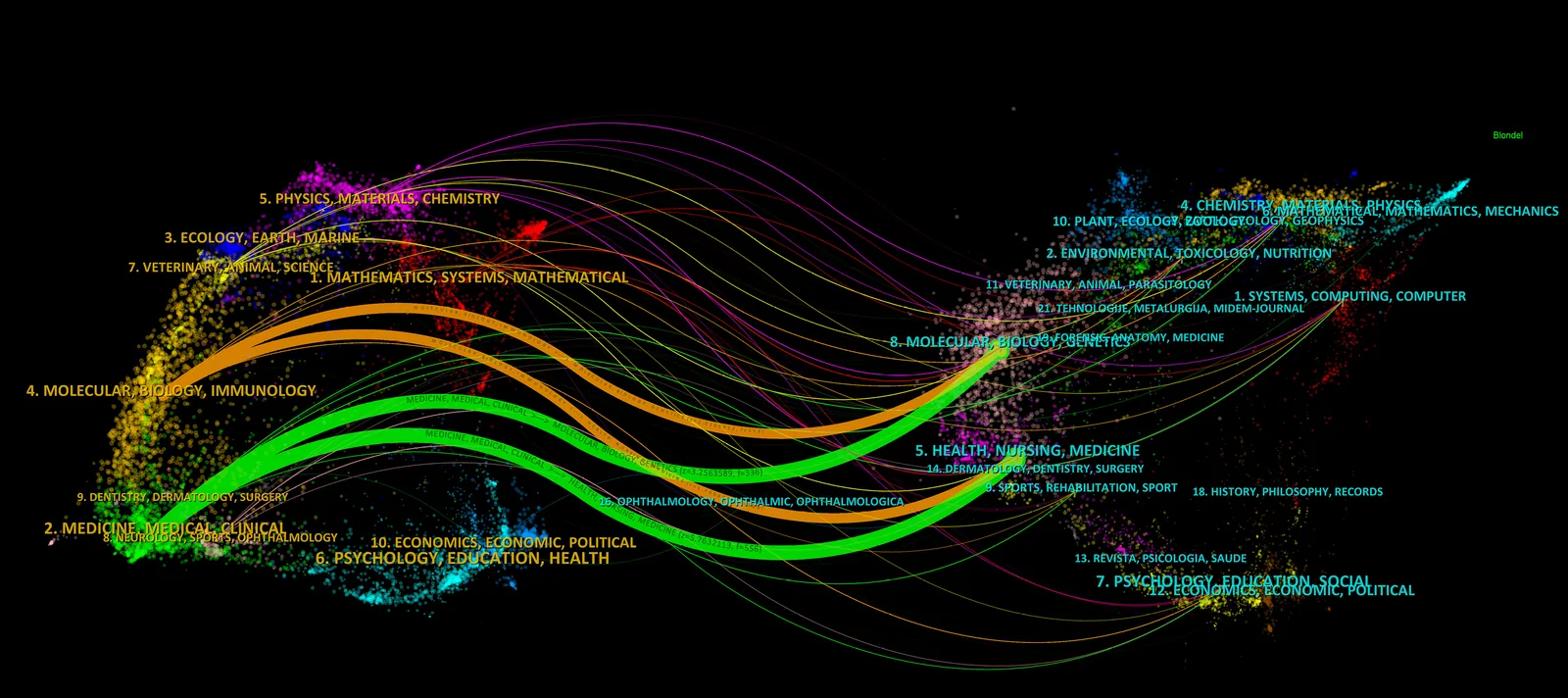
Dual-map-overlays related to machine learning in gout.

### 3.6. Analysis of keywords and citation burst analysis

Analysis of the high-centrality and high-frequency keywords revealed 333 nodes and 1303 lines (Fig. 9). According to the frequency and centrality, we found the popular keywords were “machine learning,” “uric acid,” “risk,” “association,” and “prediction” (Table 7).

**Table 7:** Top 10 frequency and centrality of keywords related to machine learning in gout.

Rank	Frequency	Keywords	Rank	Centrality	Keywords
1	63	machine learning	1	0.34	uric acid
2	52	uric acid	2	0.22	association
3	32	risk	3	0.21	ascorbic acid
4	24	association	4	0.20	risk
5	13	mortality	5	0.20	biomarkers
6	12	prediction	6	0.16	machine learning
7	12	artificial intelligence	7	0.15	diagnosis
8	11	health	8	0.10	uric acid
9	11	deep learning	9	0.10	artificial neural network
10	11	classification	10	0.09	prediction

**Figure 9.**
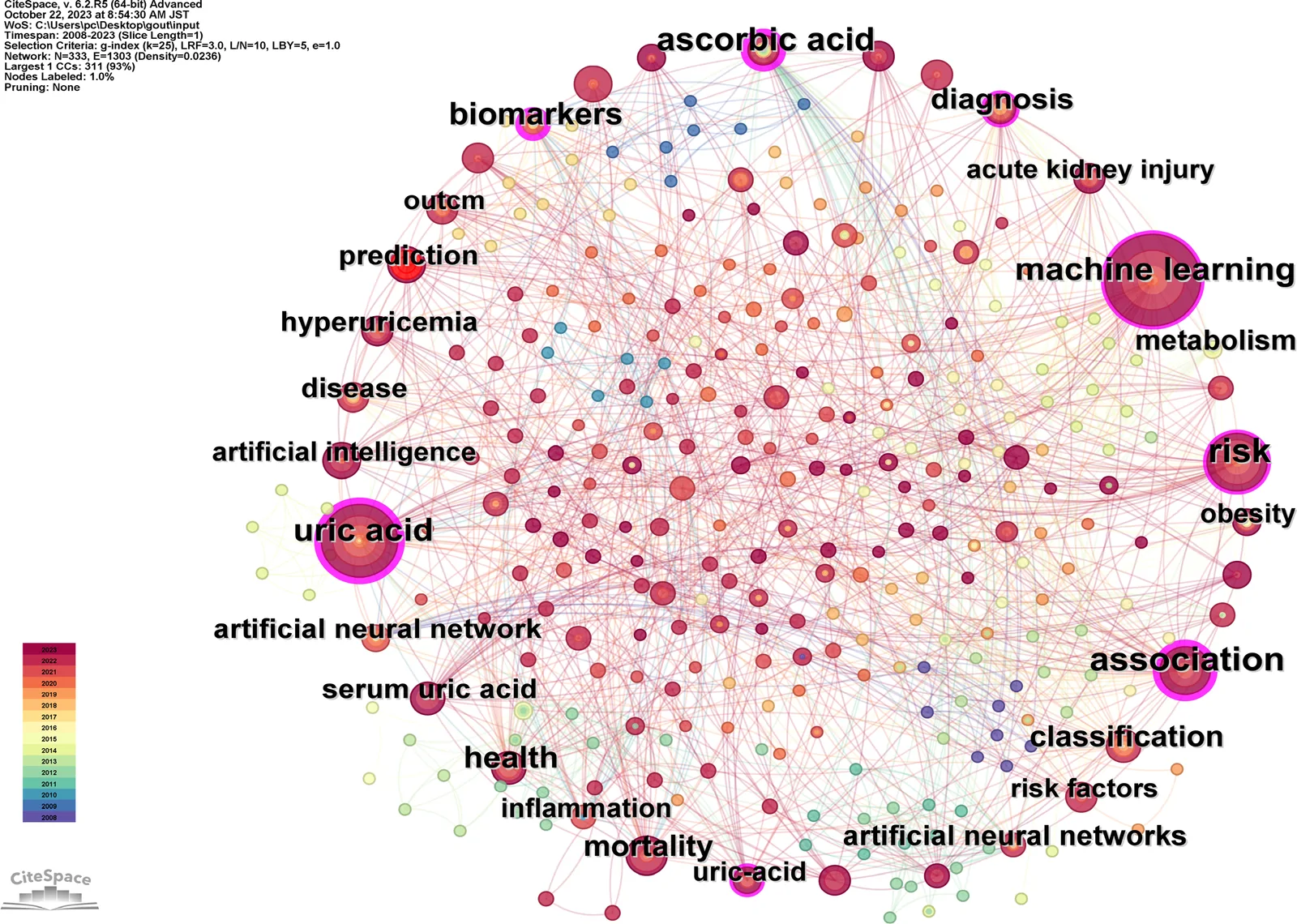
Map of keywords related to machine learning in gout.

Figure 10 displays the top 10 keywords with the strongest citation bursts in the field of machine learning in gout from 2004 to 2023. Observing the keywords with the most significant bursts over the past 20 years, it is evident that in the early years, terms like “ascorbic acid” and “classification” garnered widespread attention in the academic community. In more recent years, keywords such as “predictive models” and “deep learning” have emerged as research hotspots. Additionally, it was found that “prediction” is the keyword with the highest intensity of burst, while “ascorbic acid” possesses the longest duration of burst.

**Figure 10.**
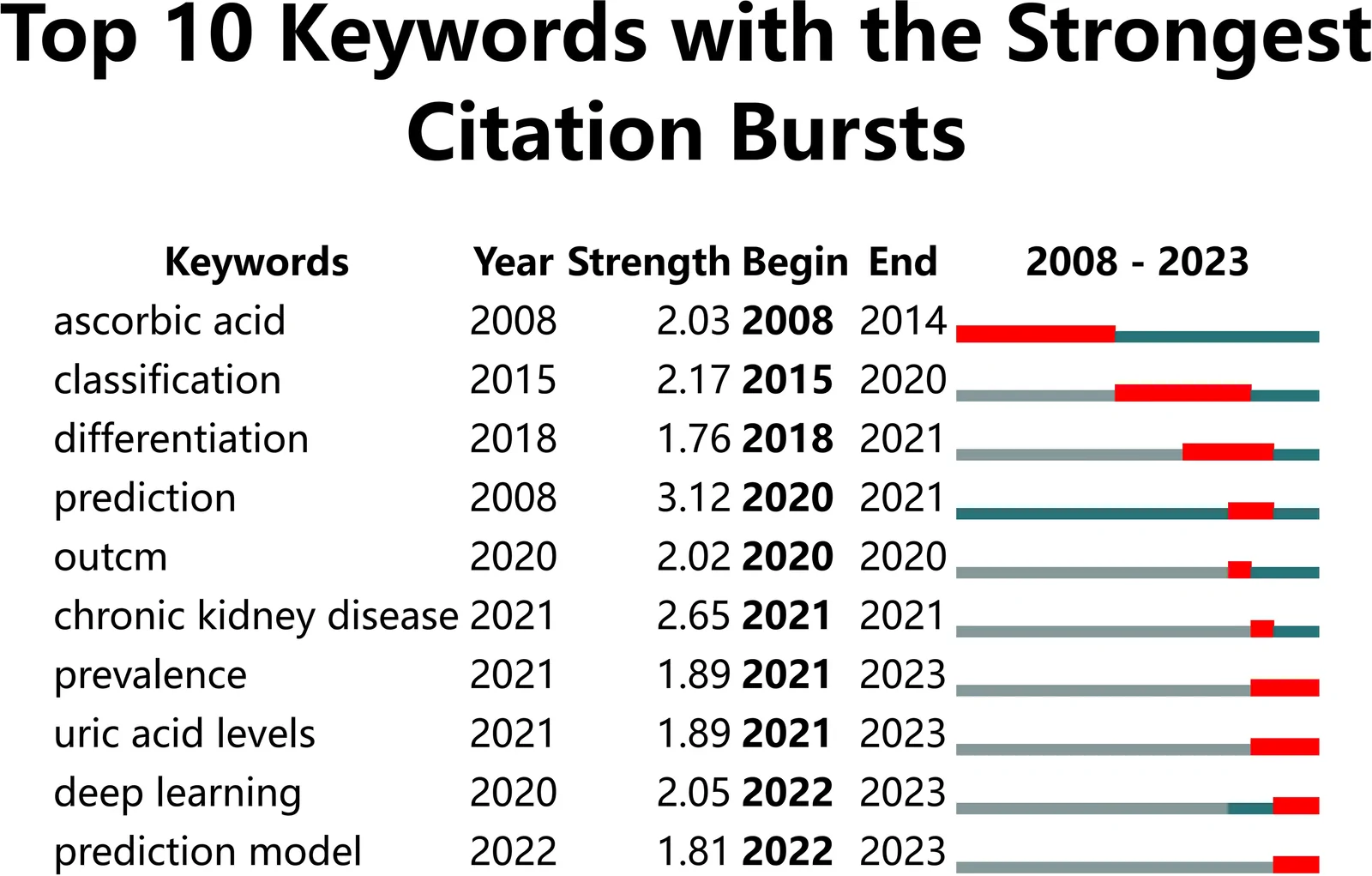
Top 10 keywords with the strongest citation bursts.

### 3.7. Keywords timeline

Figure 11 illustrates a timeline graph of keywords in the field of machine learning in gout, spanning from 2004 to 2023. Additionally, 9 key clusters of keywords were identified, labeled with numbers 0 to 8. Cluster #0, “Nitrobenzene,” focuses on artificial neural networks, ascorbic acid, and uric acid. Cluster #1, “noncommunicable Diseases,” centers on health, serum uric acid, validation, and impact. Cluster #2, “Outcomes,” emphasizes prediction, mortality, machine learning, deep learning, chronic kidney disease, acute kidney injury, artificial intelligence, and Outcome. Cluster #3, “24-hour Urine,” is concerned with risk, management, and electronic health records. Cluster #4, “Rats,” is dedicated to biomarkers and inflammation. Cluster #5, “Replication Variability,” concentrates on associations, metabolic syndrome, and cardiovascular diseases. Cluster #6, “Uric Acid Levels,” focuses on expression and predictive models. Cluster #7, “Qatar Biobank,” is devoted to obesity and classification. Finally, Cluster #8, “Machine Learning,” is focused on electrochemical detection, electrochemical biosensors, and electrocatalytic oxidation.

**Figure 11.**
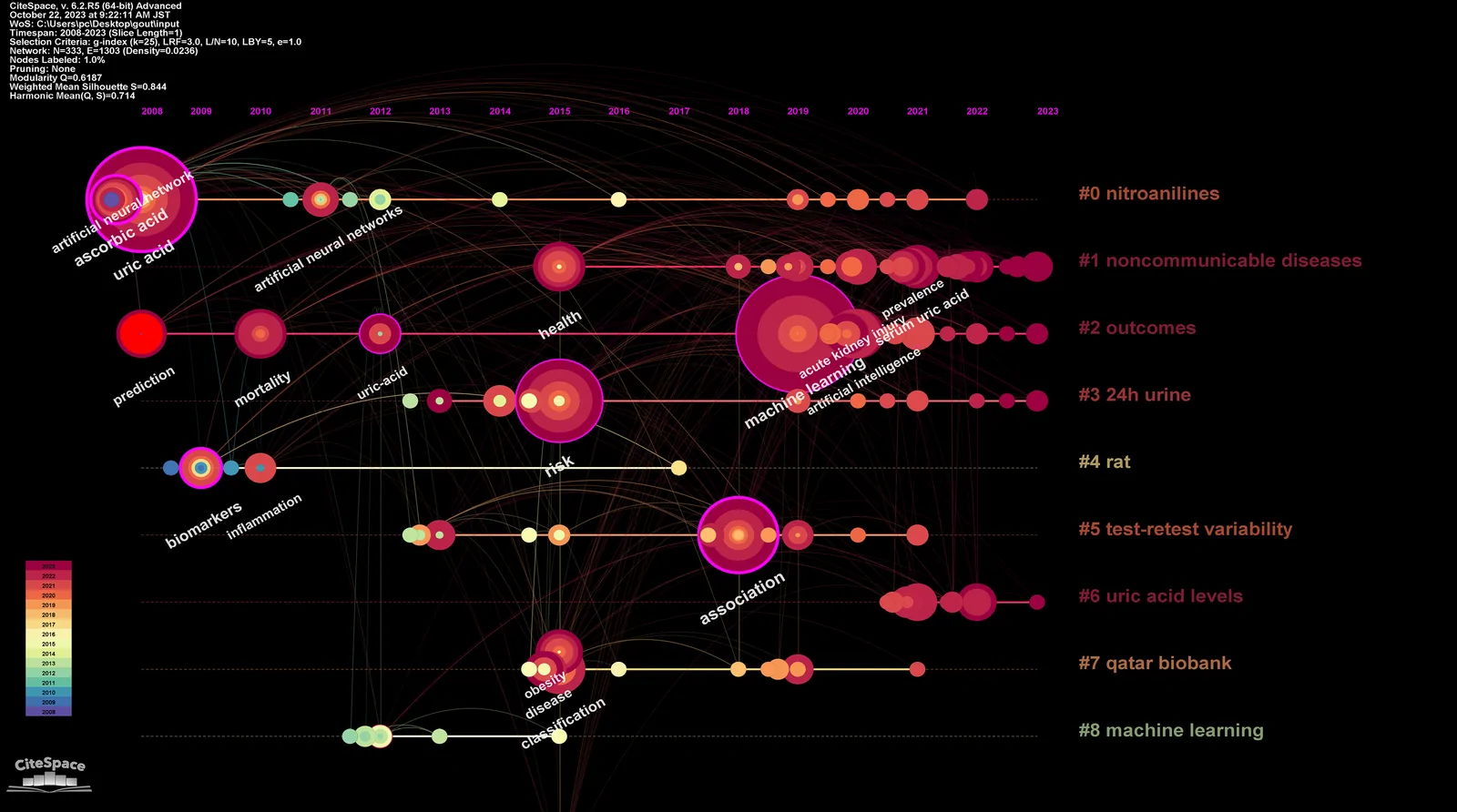
Map of keywords timeline related to machine learning in gout.

## 4. Discussion

Despite the growing use of machine learning in gout research, the field has lacked an integrated bibliometric overview of its contributors, collaboration structure, intellectual foundations, and thematic evolution. Accordingly, we analyzed 219 WoSCC articles published from 2004 to October 20, 2023. Publication activity increased markedly after 2018; China contributed the largest number of articles, whereas the US occupied a stronger bridging position in the country network; cited journals spanned multiple biomedical disciplines; and keyword analyses highlighted prediction, diagnostic models, deep learning, and algorithm selection. Together, these findings provide a reproducible map of how this research area developed and identify themes that warrant further primary research and updated bibliometric surveillance.

### 4.1. General information

The contrast between publication volume and betweenness centrality is informative: China produced the largest number of articles, whereas the US had higher centrality, suggesting a stronger bridging position among country-level collaboration paths in this dataset. This distinction cautions against equating output with network influence. The cross-disciplinary citation trajectories are consistent with the broader bibliometric literature showing that emerging biomedical technologies develop through interaction among clinical medicine, analytical science, and computational methods. In the present gout-specific corpus, the convergence of keyword frequency, citation bursts, and timeline clusters around prediction, deep learning, and model selection extends previous narrative accounts by showing when these themes became structurally prominent and how they were embedded in the literature network.

### 4.2. Research hotspots

Keywords encompass the essential content and central themes within a specific research domain. Therefore, this study aims to grasp the core themes and hotspots in the application of machine learning to the field of gout by analyzing these keywords. Throughout the research, it becomes evident that there are 2 primary focal points in the domain of gout and machine learning: the acceptance of predictive diagnostic models and the determination of the most suitable algorithmic models.

### 4.3. Acceptance of predictive diagnostic models

Early identification and accurate prediction of gout relapse bring significant benefits to patient quality of life and healthcare. Although machine learning has demonstrated its ability in medical research, its acceptance in practical clinical practice is not yet widespread.^[18–22,32]^ This lack of acceptance primarily stems from 2 concerns: the reliability of the predictive results as perceived by physicians and the issue of data privacy as perceived by patients. Jauk^[33]^ and Diprose^[34]^ conducted surveys among healthcare professionals and found that they acknowledge the convenience offered by machine learning but require repeated validation in clinical practice to enhance their trust in the results. Thamer^[35]^ suggests that many healthcare professionals may have inadequate knowledge of machine learning, and comprehensive education on artificial intelligence could positively impact their confidence and trust in this technology.

### 4.4. Determination of the most suitable algorithmic models

There are multiple existing machine learning algorithms, and new ones continue to be developed. Currently, there is no unified machine learning algorithm model for the prediction and diagnosis of gout. For instance, Shen^[24]^ combined 3 machine learning algorithms - random forest (RF), support vector machine (SVM), and logistic regression - for the early prediction of hyperuricemia. Sun^[36]^ employed stochastic gradient descent, logistic regression, and SVM for early prediction of urate. Wang^[25]^ utilized SVM and RF for predicting metabolic differences and potential biomarkers associated with frequent and rare gout attacks. Additionally, Jiang^[26]^ used 8 different machine learning models for predicting complications in gout, including logistic regression, decision trees, ensemble learning models (RF, eXtreme Gradient Boosting, light Gradient Boosting Machine, Gradient Boosting Decision Tree), SVM, and neural networks. His study revealed that sequential logistic regression and SVM achieved superior area under curve values, while RF and eXtreme Gradient Boosting models excelled in terms of recall and accuracy.

## 5. Conclusion

This bibliometric analysis indicates that research on machine learning in gout expanded markedly after 2018 and was characterized by prominent contributions from China and the US, interdisciplinary citation links, and recurring themes related to prediction, diagnostic models, and algorithm selection. These findings describe the structure and thematic evolution of the literature through October 20, 2023, rather than establishing the clinical effectiveness or future priority of any specific approach.

### 5.1. Limitations

This study has several limitations. First, only SCI-E records from WoSCC were analyzed; relevant publications indexed exclusively in Scopus, PubMed, Embase, regional databases, or preprint repositories may therefore have been missed. Second, restricting inclusion to English-language articles excluded non-English studies and other document types, such as reviews, conference proceedings, and early-access outputs, which may have introduced language and publication-type bias. Third, citation-based indicators are influenced by publication age, database indexing, and citation lag, and network structures depend partly on the selected CiteSpace parameters and pruning method. Fourth, the search ended on October 20, 2023; later publications and newly emerging themes are not represented. The findings should therefore be interpreted as a descriptive snapshot of the retrieved literature rather than a complete or current census of the field.

## Acknowledgments

The authors thank the reviewers and editorial team for their constructive comments, which helped improve the manuscript.

## Author contributions

**Conceptualization:** Xu-ming Zhang.

**Data curation:** Xu-ming Zhang, An Wu, Fang Liu.

**Investigation:** Xu-ming Zhang, An Wu, Fang Liu, Sen-guo Ji.

**Methodology:** An Wu.

**Project administration:** Sen-guo Ji.

**Resources:** Xu-ming Zhang, An Wu, Fang Liu.

**Supervision:** Sen-guo Ji.

**Software:** An Wu, Fang Liu.

**Validation:** Xu-ming Zhang.

**Visualization:** Fang Liu.

**Writing – original draft:** Xu-ming Zhang, Fang Liu, Sen-guo Ji.

**Writing – review & editing:** Xu-ming Zhang, An Wu, Fang Liu, Sen-guo Ji.
